# Discovering Vitamin-D-Deficiency-Associated Factors in Korean Adults Using KNHANES Data Based on an Integrated Analysis of Machine Learning and Statistical Techniques

**DOI:** 10.3390/nu17040618

**Published:** 2025-02-08

**Authors:** Hongryul Ahn, Seungwon Kim, Jinmyung Jung, Chan Yoon Park

**Affiliations:** 1 Division of Data Science, College of Information and Communication Technology, The University of Suwon, Hwaseong 18323, Republic of Korea; hrahn@suwon.ac.kr (H.A.); kswon0825@naver.com (S.K.); 2 Department of Food and Nutrition, The University of Suwon, Hwaseong 18323, Republic of Korea

**Keywords:** vitamin D deficiency, serum 25(OH)D levels, machine learning, Korean adults, KNHANES

## Abstract

**Background/Objectives**: Vitamin D deficiency (VDD) is a global health concern associated with metabolic disease and immune dysfunction. Despite known risk factors like limited sun exposure, diet, and lifestyle, few studies have explored these factors comprehensively on a large scale. This cross-sectional study aimed to identify VDD-associated factors in South Korea via an integrative approach of machine learning and statistical analyses using Korea National Health and Nutrition Examination Survey (KNHANES) IX-1 data. **Methods**: Using the KNHANES dataset, six machine learning algorithms were applied to evaluate VDD (serum 25[OH]D3 < 20 ng/mL)-associated factors through feature importance scores. Thereafter, multivariate linear and logistic regression models were applied to the dataset—stratified by sex and age. **Results**: Among 583 variables, 17 VDD-associated factors were identified using the CatBoost model, which achieved the highest F1 score. When these factors were assessed through statistical analysis, dietary supplement use emerged as a consistent factor associated with VDD across all subgroups (younger men, younger women, older men, and older women). In younger adults, HDL cholesterol, blood and urinary creatinine, water intake, urban residence, and breakfast frequency were significantly associated with VDD. Additionally, blood urea nitrogen and fasting plasma glucose in men and urinary sodium in women showed sex-specific associations with serum 25(OH)D levels. **Conclusions**: This study identified key VDD-associated factors in the South Korean population, which varied by age or sex. These findings highlight the multifaceted nature of VDD, influenced by dietary, lifestyle, and biochemical factors and underscore the need for strategies integrating machine learning and statistical analysis.

## 1. Introduction

Vitamin D deficiency (VDD, serum 25-hydroxyvitamin D [25(OH)D] level < 20 ng/mL) is a significant global health issue. For instance, the 2001–2018 National Health and Nutrition Examination Survey (NHANES) revealed that 24.6% of the United States (US) population is affected by VDD [1]. In South Korea, 71.4% of 21,208 participants in the 2010–2014 Korea National Health and Nutrition Examination Survey (KNHANES) had VDD [2]. Furthermore, in India, 83% of healthy adults aged 18–40 years in the Kashmir Valley reportedly had VDD [3]. VDD is well known to increase the risk of rickets and osteomalacia by regulating calcium and phosphorus balance for proper bone mineralization [4]. It has also been linked to metabolic diseases such as diabetes, cardiovascular disease, and cancer, as well as immune-related conditions like infections and autoimmune disorders [5,6,7,8]. Moreover, interest in vitamin D’s role in antiviral immunity has grown following reports of higher COVID-19 incidence in individuals with VDD [9,10].

With growing evidence linking VDD to various chronic diseases, numerous studies have investigated factors associated with VDD worldwide [11,12,13]. For example, an analysis of 55,844 Europeans using the Vitamin D Standardization Program protocol found that 13.0% had VDD, with key factors including geographical differences, sun exposure, and dietary habits [11]. A review of 15 studies involving 2833 pregnant women identified obesity, ethnicity, geographic location, and limited sun exposure as significant contributors to VDD during pregnancy [12]. Similarly, a study of 26,010 U.S. adults using NHANES 2001–2010 data reported strong associations between VDD and non-Hispanic Black race, lower educational attainment, obesity, smoking, physical inactivity, and lower milk consumption [13].

Several studies have also examined VDD risk factors in the South Korean population [2,14,15]. For example, an analysis of Korean adolescents using 2008–2009 KNHANES data identified winter season, older age, higher education level, female sex, obesity, lack of vitamin D supplementation, low milk consumption (0–<200 mL/d), and insufficient physical activity as key risk factors [14]. Another study of 21,208 Korean adults from 2010–2014 KNHANES data found associations between VDD and environmental temperature, ultraviolet radiation, walking frequency, occupation type, and shift work, with age- and sex-specific analyses revealing stronger links between VDD and low walking frequency in younger men and low educational attainment in older women [2]. Moreover, a study of 1594 South Korean nurses of childbearing age reported a high VDD prevalence (89%) and identified age, sampling month, and stress symptoms as significant risk factors [15].

Although multiple risk factors for VDD have been identified, they have not been comprehensively analyzed on a large scale, considering their diversity, ranging from eating and lifestyle habits to biochemical markers. Additionally, the KNHANES has not measured vitamin D status in recent years (2015–2021), leaving the population’s VDD status underreported. Therefore, this study utilized the most recent KNHANES IX data that contains vitamin D status, which was collected in 2022 and released in 2024. Using machine learning techniques, VDD-associated factors were screened among a variety of variables, including biochemical markers, nutrient intake, and lifestyle. Ultimately, the screened factors were statistically assessed to investigate their correlation with VDD using a dataset stratified by age and sex.

## 2. Materials and Methods

### 2.1. Data Source and Participants

**Figure 1:**
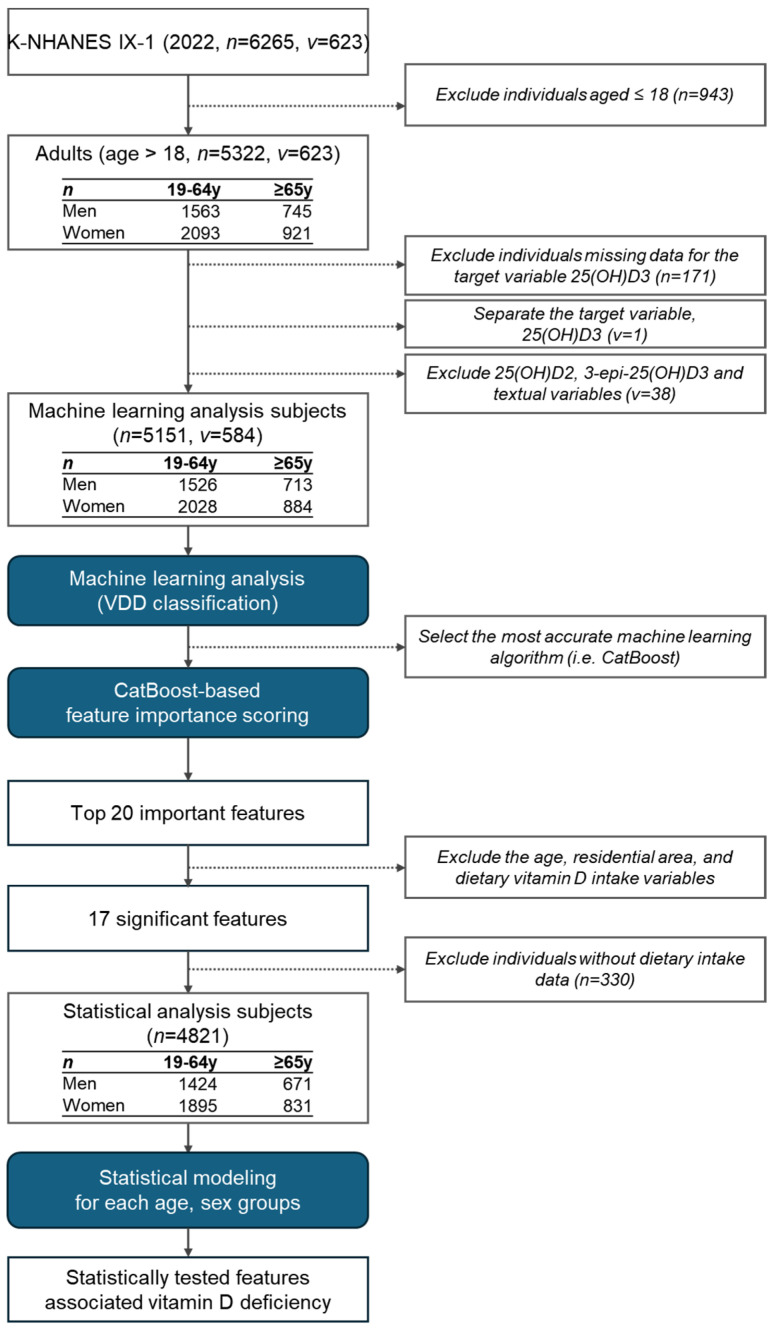
Data processing, machine learning, and statistical analysis workflow. Here, *n* denotes the number of samples and *v* represents the number of variables.

This study used data from the first round of the 2022 KNHANES IX-1 [16]. The KNHANES, a nationwide cross-sectional study conducted by the Korea Disease Control and Prevention Agency (KDCA), comprehensively assesses the health and nutritional status of the Korean population to inform national policies [17]. The KNHANES IX-1 used a two-stage stratified cluster sampling method, randomly selecting participants from 192 regions across South Korea, with 25 households chosen per region. Of the original 6265 KNHANES IX-1 participants, 943 children aged ≤ 18 years and 171 individuals without blood 25(OH)D3 data were excluded, leaving 5151 participants for the machine learning analysis. To account for potential biological differences, 330 participants without dietary intake data were excluded, and the remaining 4821 were stratified by sex and age as follows: men aged 19–64 years (*n* = 1424), women aged 19–64 years (*n* = 1895), men aged ≥ 65 years (*n* = 671), and women aged ≥ 65 years (*n* = 831) (Figure 1). The procedures included physical measurements (e.g., height, weight, waist circumference, skeletal muscle mass, and body fat), health behavior surveys (e.g., smoking, alcohol consumption, etc.), laboratory tests using blood and urine samples, and dietary intake data. The KNHANES IX-1 data collection procedure was approved by the Institutional Review Board (IRB) of the KDCA (IRB No. 2018-01-03-4C-A). The IRB of Suwon University waived ethical review and approval requirements for KNHANES IX-1 data analyses (IRB No. 2404-045-01).

### 2.2. General Characteristics

Body weight and height were measured during a health examination, and body mass index (BMI) was calculated by dividing weight in kilograms by the square of the height in meters. Data on demographic, socioeconomic, and lifestyle factors were obtained via health interviews. Household income was categorized into four quartiles: low, middle-low, middle-high, and high (Q1–Q4). Individuals who consumed alcohol at least once a month in the year preceding the interview were classified as current drinkers. Current smokers were defined as those who had smoked over 100 cigarettes in their lifetime and continued to smoke.

### 2.3. Laboratory Tests and Dietary Intake Analyses

Blood and urine samples were collected for assorted biochemical analyses. Serum vitamin D levels, including those of 25(OH)D2, 25(OH)D3, and 3-epi-25(OH)D3, were measured using liquid chromatography–mass spectrometry and expressed in ng/mL. Additional blood parameters included fasting blood glucose (mg/dL), total cholesterol (mg/dL), aspartate aminotransferase/serum glutamic–oxaloacetic transaminase (SGOT) (IU/L), hemoglobin (g/dL), blood urea nitrogen (mg/dL), blood creatinine (mg/dL), white blood cell count (Thous/µL), red blood cell count (Mil/µL), platelet count (Thous/µL), uric acid (mg/dL), and high-sensitivity C-reactive protein (mg/L). Urinary measurements encompassed urinary creatinine (mg/dL), urinary sodium (mmol/L), urinary potassium (mmol/L), and urinary albumin (µg/mL).

Dietary intake was assessed using a single 24 h dietary recall. Daily consumption of total energy and nutrients was estimated based on the KNHANES recipe and the food composition database published by the Korean Rural Development Administration [18]. The specific nutrient intake (e.g., vitamin D) used in this study was derived from the processed data provided by KNHANES.

### 2.4. Machine Learning Analyses

To prepare the machine learning dataset, we excluded variables such as 25(OH)D2, 3-epi-25(OH)D3, and textual responses, resulting in a final dataset (*n* = 5151) with 583 explanatory variables. The target variable was binary, indicating vitamin D status, classified as deficient or sufficient based on serum 25(OH)D levels. We defined the binary target using the standard cutoff of 20 ng/mL: participants with serum 25(OH)D levels ≥ 20 ng/mL were categorized as sufficient (target = 0), and those with serum 25(OH)D levels < 20 ng/mL were categorized as deficient (target = 1). This threshold aligns with the Institute of Medicine (IOM)’s definition of vitamin D deficiency [19] and is commonly used in numerous previous studies to define vitamin D deficiency and sufficiency [20,21,22,23]. Missing values were imputed via Python’s IterativeImputer from Scikit-learn (v1.3.1).

To classify VDD, six algorithms (Random Forest, GradientBoosting, XGBoost, XGBoost-RF, LightGBM, and CatBoost) were trained and validated using StratifiedShuffleSplit (50 iterations, 20% test set). Specifically, Random Forest, GradientBoosting, and StratifiedShuffleSplit were used from Scikit-learn (v1.3.1); XGBoost and XGBoost-RF from xgboost (v2.0.0); LightGBM from lightgbm (v4.1.0); and CatBoost from catboost (v1.2.2).

The performance of each algorithm was evaluated using the following metrics:

$$Precision=\frac{\mathrm{True}\;\mathrm{Positives}\;\left(\mathrm{TP}\right)}{\mathrm{True}\;\mathrm{Positives}\;\left(\mathrm{TP}\right)+\mathrm{False}\;\mathrm{Positives}\;\left(\mathrm{FP}\right)}$$

$$Sensitivity=\frac{\mathrm{True}\;\mathrm{Positives}\;\left(\mathrm{TP}\right)}{\mathrm{True}\;\mathrm{Positives}\;\left(\mathrm{TP}\right)+\mathrm{False}\;\mathrm{Negative}\;\left(\mathrm{FN}\right)}$$

$$Accuracy=\frac{\mathrm{True}\;\mathrm{Positives}\;\left(\mathrm{TP}\right)+\mathrm{True}\;\mathrm{Negative}\;\left(\mathrm{TN}\right)}{\mathrm{Total}\;\mathrm{Number}\;\mathrm{of}\;\mathrm{Samples}}$$

$$F1\;Score=2\times \frac{\mathrm{Precision}\times \mathrm{Recall}}{\mathrm{Precision}+\mathrm{Recall}}$$

$$ROC-AUC=\int_{0}^{1}\mathrm{TPR}\;\left(\mathrm{True}\;\mathrm{Positive}\;\mathrm{Rate}\right)\;d\left(\mathrm{FPR}\;\left(\mathrm{False}\;\mathrm{Positive}\;\mathrm{Rate}\right)\right)$$

For the VDD classification, CatBoost produced the highest F1 score and was thus used to compute feature importance across 50 cross-validation runs. We averaged the importance ranks from each iteration and identified 20 top factors. From these, variables directly associated with vitamin D (age, residential area, dietary vitamin D intake) were excluded, leaving 17 key factors that were subsequently validated via stratified statistical analyses.

### 2.5. Statistical Analyses

The 17 key VDD-related factors, identified through machine learning, along with general characteristics, were analyzed based on vitamin D status (deficient or sufficient). Continuous variables are presented as means ± standard errors (SE), while categorical variables are presented as percentages. The reported proportions (%) were weighted to account for the complex survey design. To assess differences between vitamin D statuses, independent t-tests and Rao–Scott chi-square tests were applied to continuous and categorical variables, respectively.

The associations between blood 25(OH)D3 level and each of the continuous variables were analyzed using multivariate linear regression models. For the categorical variables (breakfast intake frequency, dietary supplement use, and urban residence), VDD risk was evaluated using multivariate logistic regression models, with the highest breakfast intake frequency, the non-use of dietary supplements, and urban residence as the reference groups, respectively. To minimize confounding effects and account for differences in covariate distribution between vitamin-D-deficient and sufficient groups [1,24,25,26], we employed three models in both linear and logistic regression analyses: (1) the unadjusted model provided crude beta coefficients (β) and SE values for continuous variables as well as crude odds ratios (ORs) and 95% confidence intervals (CIs) for categorical variables; (2) Model 1 was adjusted for age, BMI, and total energy intake; and (3) Model 2 included additional adjustments for household income, lifestyle factors (alcohol consumption, smoking status, and regular aerobic exercise), and dietary vitamin D intake.

All statistical analyses were performed using SPSS software (version 26; IBM, Armonk, NY, USA), with adjustments made for complex survey design effects [27]. A two-sided *p*-value less than 0.05 was considered statistically significant, and exact *p*-values were reported to facilitate interpretation.

## 3. Results

**Figure 2:**
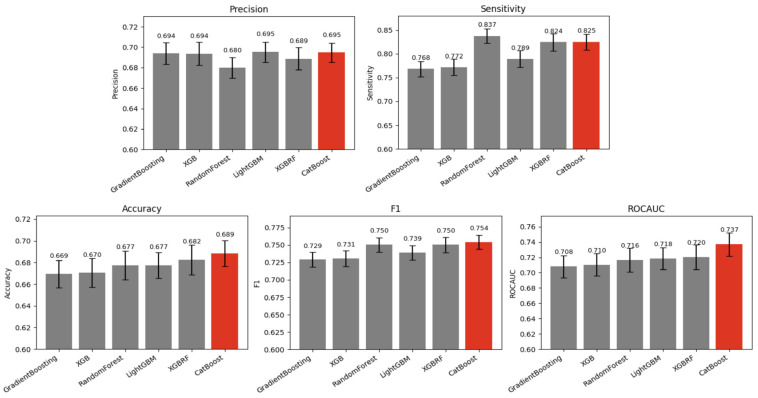
Classification accuracy of machine learning algorithms for serum vitamin D sufficiency and deficiency. The CatBoost model, highlighted in red, achieved the highest accuracy among all tested algorithms. The error bars represent the standard error of the mean.

To classify vitamin D sufficiency and deficiency, we initially trained and evaluated six tree ensemble-based algorithms using various accuracy metrics. Among these, CatBoost demonstrated the highest performance in terms of precision, accuracy, F1 score, and area under the receiver operating characteristic curve (Figure 2); therefore, it was selected as the primary model for identifying key VDD-associated variables.

**Figure 3:**
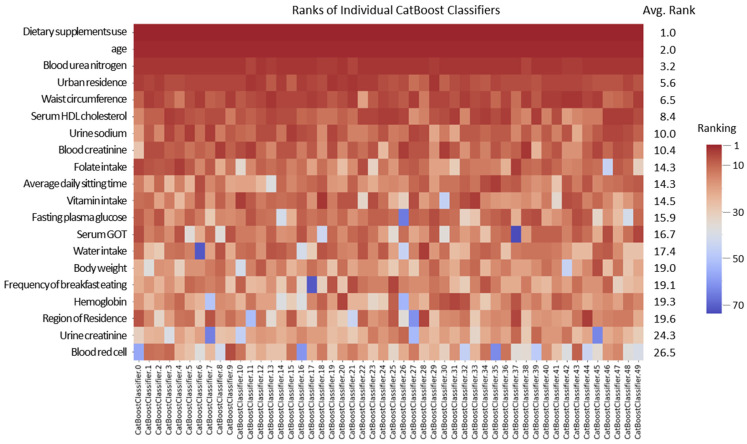
Heatmap of CatBoost-derived feature importance ranks for classifying serum vitamin D deficiency across 50 cross-validation trials. Each row corresponds to one of the top 20 most influential features, and each column represents a single cross-validation trial. The color intensity (with deeper red indicating higher importance) reflects each feature’s relative significance in that particular trial. Each number in the rightmost column indicates the average importance rank across all trials.

Using the CatBoost model, we performed 50 rounds of cross-validation to compute feature importance and subsequently averaged the rankings obtained from each iteration. This process identified 20 variables closely related to VDD from a machine learning perspective. The consistency of their rankings across the 50 iterations, visualized using a heatmap (Figure 3), confirmed the robustness of these findings. The 20 variables were dietary supplement use, age, blood urea nitrogen, urban residence, waist circumference, serum HDL cholesterol, urinary sodium, blood creatinine, folate intake, average daily sitting time, vitamin intake, fasting plasma glucose, SGOT, water intake, body weight, breakfast intake frequency, hemoglobin, region of residence, urinary creatinine, and blood red cell count. From these 20 variables, those either directly related to vitamin D or used for stratification, namely age, place of residence, and dietary vitamin D intake, were excluded, leaving 17 core variables that significantly influence serum vitamin D levels.

Thereafter, the top 17 VDD-associated variables identified through CatBoost machine learning were further analyzed by categorizing participants according to sex and age to assess differences based on vitamin D status. The percentage of participants with VDD (<20 ng/mL of serum 25[OH]D3) was 51.2%, 45.2%, 33.7%, and 22.7% in younger men (19–64 years), younger women (19–64 years), older men (≥65 years), and older women (≥65 years), respectively. Regarding general characteristics, the average age significantly varied with vitamin D status in younger men, younger women, and older men (Supplementary Tables S1 and S2). Among younger adults aged 19–64 years, the vitamin-D-sufficient group was significantly older than the vitamin-D-deficient group, while in older men, the vitamin-D-deficient group was older. Household income, BMI, and total energy intake did not significantly differ with vitamin D status across all age groups and sexes. Only in younger women were the current alcohol consumption and smoking rates significantly higher in the vitamin-D-deficient group than in the vitamin-D-sufficient group. In the vitamin-D-sufficient group, compared to those in the deficient group, women aged ≥ 65 years exhibited a higher rate of regular aerobic exercise, while men aged ≥ 65 years demonstrated higher daily vitamin D intake.

The top 17 factors for VDD, identified via CatBoost machine learning, were analyzed by comparing the vitamin D deficient and sufficient groups, categorized by sex and age (above and below 65 years) (Table 1 and Table 2). In all age and sex groups, dietary supplement use (%) was higher in the vitamin-D-sufficient group (all *p* < 0.001). Additionally, urban residence (%) was significantly more prevalent in the vitamin-D-deficient group than in the sufficient group, except for women aged ≥ 65 years. Several variables, including breakfast intake frequency, blood urea nitrogen, serum HDL cholesterol, blood creatinine, folate intake, hemoglobin, and urinary creatinine, significantly varied with vitamin D status only in younger adults aged 19–64 years, but not in older adults aged ≥ 65 years. In both younger and older men, water intake was significantly higher in the vitamin-D-sufficient group than in the deficient group.

Subsequently, associations between blood 25(OH)D3 level and each of the 14 continuous variables among the top 17 associated factors were analyzed using multivariate linear regression models (Table 3 and Supplementary Table S3). Several variables significantly associated with serum 25(OH)D level differed by age group and sex in Model 2 after adjusting for age, BMI, total energy intake, household income, alcohol consumption, smoking, aerobic exercise, and vitamin D intake. In younger adults of both sexes, serum 25(OH)D level positively correlated with serum HDL cholesterol (men: β = 0.067, SE = 0.022, *p* = 0.003; women: β = 0.041, SE = 0.019, *p* = 0.03), blood creatinine (men: β = 8.16, SE = 1.95, *p* < 0.001; women: β = 5.19, SE = 2.27, *p* = 0.02), and water intake (men: β = 0.001, SE = 0.001, *p* = 0.02; women: β = 0.002, SE = 0.001, *p* = 0.02) but negatively correlated with urinary creatinine (men: β = −0.009, SE = 0.003, *p* = 0.002; women: β = −0.014, SE = 0.004, *p* = 0.001). In men across all age groups, blood urea nitrogen (19–64 years: β = 0.398, SE = 0.075; ≥65 years: β = 0.313, SE = 0.109) and water intake (19–64 years: β = 0.001, SE = 0.001; ≥65 years: β = 0.003, SE = 0.001) exhibited significantly positive associations with serum 25(OH)D level, while fasting plasma glucose displayed a negative association (19–64 years: β = −0.022, SE = 0.008, *p* = 0.006; ≥65 years: β = −0.033, SE = 0.012, *p* = 0.009). On the other hand, urinary sodium and creatinine levels yielded significantly negative correlations with serum 25(OH)D levels in both younger and older women.

**Figure 4:**
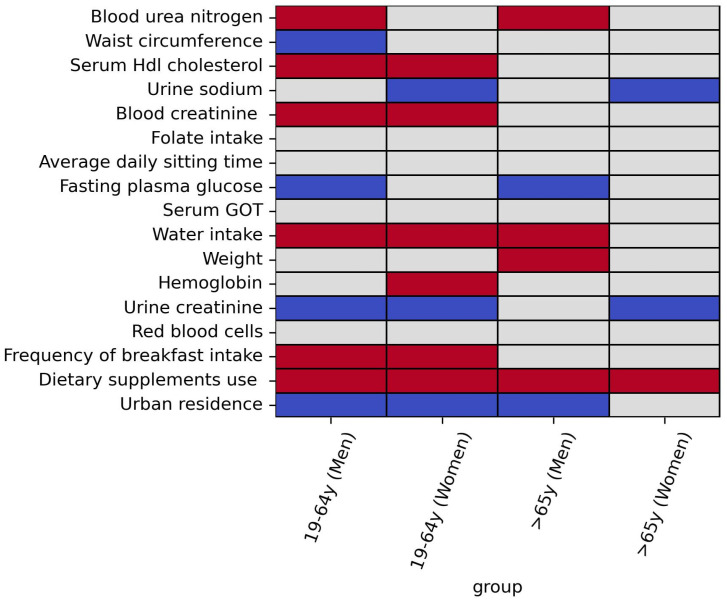
Heatmap representing the significance of the association with vitamin D level outlined in Table 3 and Table 4. The gray color indicates no significant association. The blue and red colors represent significantly negative and positive associations, respectively. Table 4 describes the risk of VDD, rather than its associations with vitamin D levels, for the three variables (i.e., breakfast intake frequency, dietary supplement intake, and rural residence); however, for consistency, the heatmap illustrates these variables’ associations with vitamin D level.

Multivariate logistic regression models were used to ascertain whether each of the three categorical variables among the top 17 factors was associated with VDD according to sex and age (Table 4). In Model 2 (adjusted for confounding factors), the non-use of dietary supplements had significantly higher odds of VDD than supplement use in younger men (OR, 3.13; 95% CI, 2.23–4.41), younger women (OR, 3.71; 95% CI, 2.75–4.99), older men (OR, 3.26; 95% CI, 2.11–5.03), and older women (OR, 2.31; 95% CI, 1.51–3.54). In addition, urban residence (%) exhibited significantly higher odds of VDD than rural residence in younger men (OR, 1.94; 95% CI, 1.19–3.16), younger women (OR, 2.00; 95% CI, 1.26–3.18), and older men (OR, 2.88; 95% CI, 1.67–4.97). In younger adults of both sexes, consuming breakfast less than once a week or 1–2 times a week was associated with significantly higher odds of VDD than consuming breakfast 5–7 times a week. However, older adults did not display a significant association between breakfast intake frequency and VDD. A summarized heatmap of the results outlined in Table 3 and Table 4 is shown in Figure 4.

## 4. Discussion

Our findings reveal that VDD remains a significant public health concern in South Korea, particularly among younger adults (45.2–51.2%). Using the most recent KNHANES IX-1 data, this study identified several novel VDD-associated factors in the Korean population. Machine learning techniques initially screened the top 17 factors, which were then further analyzed by age and sex using statistical methods. Among these, dietary supplement use showed a consistent and strong association with VDD across all age and sex groups. In younger adults of both sexes, HDL cholesterol, blood and urinary creatinine, water intake, urban residence, and breakfast intake frequency were significantly linked to VDD. Additionally, several factors, including blood urea nitrogen and fasting plasma glucose in men and urinary sodium in women, were associated with serum 25(OH)D levels in a sex-dependent manner.

Machine learning has increasingly been applied in health data analytics, demonstrating its capability to uncover complex relationships and improve diagnostic accuracy across diverse health domains. Several studies have applied machine learning techniques to cross-sectional models using KNHANES data to identify factors associated with specific diseases [28,29,30,31]. For instance, metabolic syndrome studies utilizing deep learning and ensemble methods have identified key contributors such as dietary intake, triglycerides, and waist-to-height ratio [28,29]. Similarly, machine learning analyses of cardiovascular disease risk factors, including LightGBM and XGBoost, have highlighted age, hypertension, and BMI as major determinants [30]. Moreover, machine learning has been applied to conditions such as osteoporosis and obesity, demonstrating its capacity to integrate biochemical, lifestyle, and genetic data, uncover complex associations, and provide stratified insights into health conditions [28,31]. In the context of VDD, most studies have primarily relied on traditional statistical methods [32,33,34], with limited application of machine learning. A recent study on VDD using NHANES data applied machine learning techniques such as Gradient Boosting Machines, Neural Networks, and XGBoost; however, it focused on classifying and predicting VDD rather than finding VDD-related factors [35]. Therefore, this study may be the first to integrate machine learning and statistical methods to comprehensively identify key VDD-related factors, offering a deeper understanding of its determinants.

In this study, the non-use of dietary supplements emerged as the most significant factor associated with VDD across all sex and age subgroups. Regarding dietary supplements use, the KNHANES IX-1 assessed experiences of taking supplements for >2 weeks within the preceding year, without detailed investigation into specific supplement use. Therefore, accurately assessing vitamin D intake from supplement use proves challenging; however, among Koreans, the associations with VDD might be attributable to considerable vitamin D intake via dietary supplements. Several previous studies have established vitamin D as a top dietary supplement consumed by Koreans [36,37]. According to an analysis based on 2015 KNHANES data, multivitamin mineral supplements constituted the most frequently consumed dietary supplement in both men and women, with a rate of 89.6 per 1000 people, while vitamin D ranked seventh [37]. As numerous multivitamin supplements contain vitamin D, cumulative intake from multiple dietary supplements has been reported as a potential reason for exceeding the upper limit of vitamin D [38]. Additionally, a study on Koreans aged >10 years found that the elderly had the highest serum 25(OH)D levels, despite their low dietary vitamin D intake, suggesting supplementation as a contributing factor [34,39]. In particular, vitamin D was the most frequently supplemented nutrient during the first and second waves of the COVID-19 pandemic owing to its beneficial role in supporting immune defense against viral infections [10,40]. Therefore, the close association between VDD and dietary supplement use in this study is potentially attributable to high vitamin-D-related supplement intake in the Korean population.

Interestingly, certain blood factors were significantly associated with vitamin D only in younger adults, including its positive correlation with serum HDL cholesterol, aligning with previous findings [41,42,43]. A study analyzing 28,084 NHANES adults aged 20–59 years or yielded a significantly positive correlation between HDL cholesterol and vitamin D (25[OH]D) level [41]. This positive association between HDL cholesterol and vitamin D levels may be related to the protective effects of HDL cholesterol on cardiovascular health, suggesting that vitamin D potentially supports cardiovascular health through its role in lipid metabolism. This highlights the potential cardiovascular benefits of maintaining optimal vitamin D levels [43].

The current study also found younger adults residing in rural areas to have higher vitamin D levels than their urban counterparts, possibly because of greater outdoor activity in rural settings, resulting in more sunlight exposure and, therefore, higher vitamin D synthesis. Wakayo et al. [44] reported a higher prevalence of vitamin D deficiency in urban (61.8%) than rural (21.2%) Ethiopian schoolchildren. However, a study in western Ireland found lower serum 25(OH)D levels in rural adults year-round [45], likely due to geographic differences. In addition, we found an association between breakfast intake and vitamin D in younger adults, consistent with previous studies. Research in Brazilian adolescents and US Army recruits highlighted that regular breakfast consumption improves vitamin D levels and reduces VDD risk [46,47].

Creatinine, a byproduct of creatine phosphate breakdown in muscles, is a marker of skeletal muscle mass under stable kidney function [48,49]. When kidney function declines, blood creatinine levels increase, while urinary creatinine decreases due to reduced filtration capacity [50]. In younger adults, blood creatinine positively correlated with serum 25(OH)D, whereas urinary creatinine showed a negative correlation. Vitamin D has been linked to kidney function, with studies reporting its negative association with albuminuria and potential benefits in CKD management [51,52]. Moreover, vitamin D may influence muscle creatinine production, as clinical trials have shown increased serum creatinine levels following vitamin D receptor activation, independent of kidney function [53,54].

Several VDD-associated variables, such as blood urea nitrogen, fasting plasma glucose, and urinary sodium exhibited sex-specific differences. As blood urea nitrogen and urinary sodium reflect kidney health, these findings suggest a potential link between vitamin D status and kidney function. Furthermore, fasting glucose and urinary sodium are related to type 2 diabetes and hypertension, conditions in which vitamin D reportedly influences insulin secretion, sensitivity, and β-cell function while exerting anti-inflammatory effects [7]. Further research is needed to explore these sex-specific associations and potential therapeutic implications. Lastly, dietary folate intake showed no significant association with VDD when stratified by sex and age, despite being identified as a key factor by the CatBoost model. This discrepancy may result from interaction effects or reduced statistical power due to stratification. The “vitamin D–folate hypothesis” suggests an evolutionary link between skin pigmentation, vitamin D synthesis, and folate preservation, as UVR both stimulates vitamin D production and degrades folate. Additionally, in vitro studies indicate that vitamin D upregulates folate transporters, further supporting this interplay [55,56,57]. While these findings imply a potential connection between vitamin D and folate, further research is needed to clarify their relationship.

This study has several limitations. First, its cross-sectional design prevents causal inferences, and reverse causation cannot be ruled out. Second, key determinants of 25(OH)D levels, such as sun exposure time and seasonal effects [58,59,60,61,62], were not adjusted for due to data limitations in KNHANES IX-1. While we indirectly assessed sun exposure by comparing urban and rural residents, this approach does not fully capture its effects. Third, we used a standard cutoff to classify participants as vitamin D deficiency as <20 ng/mL [19], defining groups by a relatively narrow margin may skew causative interpretations. Fourth, self-reported lifestyle factors may introduce recall bias despite the availability of objective laboratory data. Fifth, key variables like medication use and surgical history lacked detail, limiting further analysis. Sixth, the dataset does not specify supplement type or dosage, preventing precise evaluation of their impact on vitamin D levels. Lastly, the study may be subject to statistical power limitations (Type II error), particularly for older age groups with a smaller sample size, potentially affecting the detection of significant associations in stratified analyses. Despite these limitations, this study provides a comprehensive analysis of VDD-related factors in a large national sample, leveraging machine learning for robust feature selection. Future research should incorporate sun exposure, seasonal variations, alternative criteria for differentiating between vitamin D deficiency and sufficiency, supplement details, and detailed dietary assessments for a more precise understanding of VDD.

## 5. Conclusions

VDD remains a prevalent health issue in South Korea. Through an integrative approach combining machine learning and statistical analyses, several key factors associated with VDD were identified, including dietary supplement use, urinary creatinine levels, urban residence, and breakfast frequency. In this study, machine learning models capable of handling complex correlations among variables were employed to screen the most relevant factors, facilitating the analysis of a comprehensive set of 583 variables. As bioanalytical and data processing technologies continue to advance, future research will need to consider an even greater number of factors, further emphasizing the importance of machine learning approach. Furthermore, the inclusion of new data sources (i.e., KNHANES IX-1) and the implementation of age- and sex-stratified statistical analyses are also key strengths of this study. This research highlights the significance of integrating machine learning with statistical analysis, demonstrating its potential for more comprehensive and insightful investigations.

## Figures and Tables

**Table 1:** Characteristics of the 17 variables identified through machine learning in participants aged 19–64 years.

	Men (*n* = 1424)			Women (*n* = 1895)		
	VD Deficient (*n* = 729)	VD Sufficient (*n* = 695)	*p* Value *	VD Deficient (*n* = 857)	VD Sufficient (*n* = 1038)	*p* Value *
Breakfast intake frequency, %			<0.001			<0.001
5–7 times/wk	261 (33)	393 (52)		310 (33)	583 (54)	
3–4 times/wk	96 (14)	78 (13)		121 (14)	138 (13)	
1–2 times/wk	109 (16)	75 (12)		172 (21)	119 (11)	
<1 time/wk	263 (37)	149 (23)		254 (32)	198 (21)	
Dietary supplement use, %	390 (53)	528 (78)	<0.001	550 (63)	916 (89)	<0.001
Urban residence, %	632 (90)	528 (81)	<0.001	750 (91)	835 (85)	0.007
Blood urea nitrogen	13.4 ± 0.1	14.6 ± 0.1	<0.001	12.2 ± 0.1	13.4 ± 0.2	<0.001
Waist circumference, cm	89.1 ± 0.4	88.3 ± 0.4	0.13	77.8 ± 0.5	77.8 ± 0.4	0.96
Serum HDL cholesterol	50.6 ± 0.5	52.2 ± 0.6	0.03	63.7 ± 0.7	65.4 ± 0.5	0.04
Urinary sodium	115 ± 2	113 ± 2	0.45	104 ± 2	99.2 ± 1.8	0.06
Blood creatinine	0.91 ± 0.01	0.93 ± 0.01	0.005	0.67 ± 0.0	0.07 ± 0.01	0.006
Folate intake	314 ± 5	338 ± 7	0.006	264 ± 6	286 ± 6	0.005
Average daily sitting time, h	14.4 ± 0.8	15.9 ± 1.2	0.07	14.0 ± 1.0	14.0 ± 0.7	0.33
Fasting plasma glucose	102 ± 1	102 ± 1	0.74	95.4 ± 0.7	95.7 ± 0.8	0.76
SGOT	24.4 ± 0.6	24.8 ± 0.6	0.59	18.8 ± 0.4	20.3 ± 0.3	0.001
Water intake	1103 ± 26	1212 ± 26	0.002	950 ± 21	988 ± 19	0.14
Body weight	76.5 ± 0.5	75.3 ± 0.5	0.09	60.4 ± 0.5	58.5 ± 0.4	0.002
Hemoglobin	15.3 ± 0.0	15.2 ± 0.0	0.008	12.9 ± 0.0	13.1 ± 0.0	<0.001
Urinary creatinine	183 ± 4	160 ± 4	<0.001	142 ± 3	114 ± 3	<0.001
Red blood cell count	5.1 ± 0.0	5.0 ± 0.0	<0.001	4.4 ± 0.0	4.4 ± 0.0	0.07

Data are expressed as the mean ± SE for continuous variables or numbers (%) for categorical variables. The reported proportions (%) were weighted to account for the complex survey design. * Differences were determined via ANOVA for continuous variables or Rao–Scott chi-square tests for categorical variables. ANOVA, analysis of variance; HDL, high-density lipoprotein; SE, standard error; SGOT, serum glutamic–oxaloacetic transaminase; VD, vitamin D.

**Table 2:** Characteristics of the 17 variables identified through machine learning in participants aged ≥ 65 years.

	Men (*n* = 671)			Women (*n* = 831)		
	VD Deficient (*n* = 226)	VD Sufficient (*n* = 445)	*p* Value *	VD Deficient (*n* = 222)	VD Sufficient (*n* = 609)	*p* Value *
Breakfast intake frequency, %			0.68			0.75
5–7 times/wk	211 (94)	419 (93)		197 (90)	545 (89)	
3–4 times/wk	3 (1)	7 (3)		7 (3)	21 (4)	
1–2 times/wk	5 (2)	4 (1)		6 (2)	18 (3)	
<1 time/wk	7 (3)	15 (4)		12 (5)	25 (4)	
Dietary supplement use, %	92 (44)	304 (70)	<0.001	124 (57)	467 (78)	<0.001
Urban residence, %	176 (84)	266 (70)	0.01	153 (78)	386 (74)	0.35
Blood urea nitrogen	16.9 ± 0.4	17.5 ± 0.3	0.15	16.7 ± 0.4	16.7 ± 0.3	1.00
Waist circumference, cm	89.5 ± 0.6	88.8 ± 0.5	0.34	86.9 ± 0.8	84.7 ± 0.5	0.02
Serum HDL cholesterol	50.5 ± 0.9	52.8 ± 0.8	0.052	57.1 ± 1.1	58.3 ± 0.7	0.35
Urinary sodium	125 ± 4	121 ± 3	0.48	119 ± 4	109 ± 2	0.03
Blood creatinine	1.01 ± 0.02	0.96 ± 0.01	0.06	0.74 ± 0.02	0.73 ± 0.01	0.28
Folate intake	379 ± 12	377 ± 9	0.91	318 ± 13	311 ± 10	0.63
Average daily sitting time, h	19.0 ± 1.9	16.2 ± 1.6	0.76	27.0 ± 3.0	18.8 ± 1.4	0.02
Fasting plasma glucose	113 ± 3	107 ± 1	0.02	108 ± 2	104 ± 1	0.07
SGOT	23.6 ± 0.8	24.5 ± 0.5	0.32	23.2 ± 0.5	24.2 ± 0.5	0.18
Water intake	856 ± 31	964 ± 33	0.02	744 ± 35	769 ± 25	0.52
Body weight	66.1 ± 0.7	66.7 ± 0.5	0.51	58.1 ± 0.8	56.9 ± 0.4	0.21
Hemoglobin	14.2 ± 0.1	14.2 ± 0.1	0.88	12.8 ± 0.1	12.9 ± 0.05	0.29
Urinary creatinine	104 ± 4	109 ± 3	0.50	79.7 ± 3.6	72.5 ± 1.9	0.08
Red blood cell count	1.59 ± 0.03	4.58 ± 0.02	0.65	4.23 ± 0.03	4.24 ± 0.02	0.80

Data are expressed as the mean ± SE for continuous variables or numbers (%) for categorical variables. The reported proportions (%) were weighted to account for the complex survey design. * Differences were determined via ANOVA for continuous variables or Rao–Scott chi-square tests for categorical variables. ANOVA, analysis of variance; HDL, high-density lipoprotein; SE, standard error; SGOT, serum glutamic–oxaloacetic transaminase; VD, vitamin D.

**Table 3:** Association between serum 25(OH)D3 level and 14 VDD-associated continuous variables identified through machine learning by age and sex.

Continuous Variables	19–64 Years	Model 1 *		Model 2 ^†^		≥65 Years	Model 1 *		Model 2 ^†^	
		β	*p*-Value	β	*p*-Value		β	*p*-Value	β	*p*-Value
Blood urea nitrogen	Men	0.386	<0.001	0.398	<0.001	Men	0.297	<0.001	0.313	0.005
	Women	0.105	0.16	0.121	0.09	Women	0.179	0.03	0.160	0.06
Waist circumference	Men	−0.185	0.008	−0.190	0.01	Men	0.012	0.92	0.040	0.72
	Women	−0.048	0.56	−0.028	0.75	Women	−0.077	0.52	−0.116	0.39
Serum HDL cholesterol	Men	0.075	0.001	0.067	0.003	Men	0.041	0.25	0.044	0.25
	Women	0.042	0.02	0.041	0.03	Women	0.071	0.09	0.052	0.18
Urinary sodium	Men	−0.010	0.04	−0.009	0.08	Men	−0.016	0.12	−0.016	0.15
	Women	−0.017	0.004	−0.015	0.005	Women	−0.037	0.002	−0.037	0.004
Blood creatinine	Men	8.16	<0.001	8.16	<0.001	Men	−1.926	0.34	−1.97	0.31
	Women	4.93	0.04	5.19	0.02	Women	1.286	0.65	1.17	0.68
Folate intake	Men	0.002	0.32	0.001	0.62	Men	−0.001	0.82	−0.001	0.68
	Women	0.001	0.55	0.002	0.49	Women	−0.001	0.89	−0.001	0.79
Average daily sitting time	Men	0.010	0.37	0.002	0.98	Men	−0.016	0.42	−0.065	0.09
	Women	0.015	0.27	−0.061	0.34	Women	−0.026	0.24	−0.032	0.50
Fasting plasma glucose	Men	−0.023	0.003	−0.022	0.006	Men	−0.035	0.006	−0.033	0.009
	Women	−0.018	0.20	−0.012	0.39	Women	−0.008	0.75	−0.000	1.00
SGOT	Men	0.009	0.56	−0.004	0.79	Men	0.012	0.83	−0.008	0.89
	Women	0.039	0.14	0.032	0.18	Women	0.035	0.45	0.036	0.42
Water intake	Men	0.002	0.003	0.001	0.02	Men	0.003	0.006	0.003	0.02
	Women	0.001	0.02	0.002	0.02	Women	0.002	0.25	0.002	0.27
Weight	Men	0.086	0.06	0.084	0.08	Men	0.163	0.08	0.209	0.02
	Women	0.007	0.90	0.009	0.90	Women	0.047	0.70	−0.064	0.61
Hemoglobin	Men	−0.364	0.14	−0.280	0.26	Men	0.152	0.72	0.055	0.89
	Women	0.778	0.002	0.791	0.003	Women	0.157	0.73	0.392	0.43
Urinary creatinine	Men	−0.010	0.001	−0.009	0.002	Men	−0.009	0.26	−0.007	0.38
	Women	−0.014	<0.001	−0.014	0.001	Women	−0.029	0.009	−0.036	0.001
Red blood cells	Men	−0.973	0.18	−0.762	0.31	Men	−0.120	0.92	−0.228	0.84
	Women	−0.536	0.54	−0.412	0.65	Women	−0.414	0.75	0.398	0.77

This analysis was conducted using multiple regression analysis between serum 25(OH)D3 level and each variable identified through machine learning with adjustment. A table presenting the standard errors of β is prepared in Supplementary Table S3. * Model 1: adjusted for age, body mass index, and total energy intake. ^†^ Model 2: model 1 plus adjustments for household income, alcohol consumption, smoking, aerobic exercise, and vitamin D intake. 25(OH)D3, 25-hydroxyvitamin D3; HDL, high-density lipoprotein; SGOT, serum glutamic–oxaloacetic transaminase; VDD, vitamin D deficiency.

**Table 4:** Risk of VDD according to each VDD-associated categorical variable (breakfast intake frequency, dietary supplement intake, or urban residence) identified through machine learning by age and sex.

Categorical Variables	Sex (Age)		Model 1 *		Model 2 ^†^	
			OR	95% CI	OR	95% CI
Breakfast intake frequency	Men (19–64)	<1 time/wk	2.07	1.53, 2.79	1.89	1.38, 2.59
		1–2 times/wk	1.76	1.16, 2.66	1.65	1.09, 2.51
		3–4 times/wk	1.30	0.88, 1.92	1.25	0.84, 1.85
		5–7 times/wk	1 (ref)		1 (ref)	
	Women (19–64)	<1 time/wk	1.61	1.22, 2.11	1.47	1.11, 1.96
		1–2 times/wk	2.04	1.42, 2.95	2.02	1.36, 2.99
		3–4 times/wk	1.27	0.92, 1.76	1.23	0.88, 1.72
		5–7 times/wk	1 (ref)		1 (ref)	
	Men (≥65)	<1 time/wk	0.82	0.30, 2.26	0.92	0.31, 2.69
		1–2 times/wk	2.31	0.52, 10.2	2.19	0.56, 8.52
		3–4 times/wk	0.74	0.17, 3.25	0.79	0.17, 3.64
		5–7 times/wk	1 (ref)		1 (ref)	
	Women (≥65)	<1 time/wk	1.15	0.56, 2.38	1.09	0.48, 2.46
		1–2 times/wk	0.73	0.25, 2.15	0.76	0.24, 2.37
		3–4 times/wk	0.63	0.21, 1.89	0.82	0.27, 2.50
		5–7 times/wk	1 (ref)		1 (ref)	
Dietary supplement use	Men (19–64)	No	3.06	2.21, 4.23	3.13	2.23, 4.41
		Yes	1 (ref)		1 (ref)	
	Women (19–64)	No	3.90	2.94, 5.16	3.71	2.75, 4.99
		Yes	1 (ref)		1 (ref)	
	Men (≥65)	No	2.91	1.91, 4.43	3.26	2.11, 5.03
		Yes	1 (ref)		1 (ref)	
	Women (≥65)	No	2.77	1.90, 4.05	2.31	1.51, 3.54
		Yes	1 (ref)		1 (ref)	
Urban residence	Men (19–64)	Urban	1.80	1.11, 2.90	1.94	1.19, 3.16
		Rural	1 (ref)		1 (ref)	
	Women (19–64)	Urban	1.67	1.06, 2.62	2.00	1.26, 3.18
		Rural	1 (ref)		1 (ref)	
	Men (≥65)	Urban	2.33	1.36, 4.02	2.88	1.67, 4.97
		Rural	1 (ref)		1 (ref)	
	Women (≥65)	Urban	1.26	0.92, 1.74	1.26	0.86, 1.84
		Rural	1 (ref)		1 (ref)	

This analysis was conducted using multivariate logistic regression models to assess the risk of VDD with adjustments. * Model 1: adjusted for age, body mass index, and total energy intake. ^†^ Model 2: model 1 plus adjustments for household income, alcohol consumption, smoking, aerobic exercise, and vitamin D intake. CI, confidence interval; OR, odds ratio; VDD, vitamin D deficiency.

## Data Availability

The data (2022 KNHANES IX-1) presented in this study are available at https://knhanes.kdca.go.kr/knhanes/main.do (accessed on 30 January 2025).

## Supplementary Materials

The following supporting information can be downloaded at: https://www.mdpi.com/article/10.3390/nu17040618/s1, Table S1: General characteristics of the participants aged 19–64 y, Table S2: General characteristics of the participants aged ≥65 y, Table S3: Table presenting the standard errors of beta in Table 3.

## Abbreviations

25(OH)D: 25-hydroxyvitamin D; BMI: body mass index; CIs: confidence intervals; CKD: chronic kidney disease; COVID-19: coronavirus disease 2019; HDL: high-density lipoprotein; IRB: Institutional Review Board; KNHANES: Korea National Health and Nutrition Examination Survey; NHANES: National Health and Nutrition Examination Survey; OR: odds ratios; SE: standard error; SGOT: serum glutamic–oxaloacetic transaminase; US: United States; VDD: Vitamin D deficiency.

## References

[1] Cui A., Xiao P., Ma Y., Fan Z., Zhou F., Zheng J., Zhang L. (2022). Prevalence, trend, and predictor analyses of vitamin D deficiency in the US population, 2001–2018. Front. Nutr..

[2] Park H.Y., Lim Y.-H., Park J.B., Rhie J., Lee S.-J. (2020). Environmental and occupation factors associated with vitamin D deficiency in Korean adults: The Korea National Health and Nutrition Examination Survey (KNHANES) 2010–2014. Int. J. Environ. Res. Public Health.

[3] Zargar A., Ahmad S., Masoodi S., Wani A., Bashir M., Laway B., Shah Z. (2007). Vitamin D status in apparently healthy adults in Kashmir Valley of Indian subcontinent. Postgrad. Med. J..

[4] Minisola S., Colangelo L., Pepe J., Diacinti D., Cipriani C., Rao S.D. (2021). Osteomalacia and vitamin D status: A clinical update 2020. J. Bone Miner. Res. Plus.

[5] Holick M.F. (2017). The vitamin D deficiency pandemic: Approaches for diagnosis, treatment and prevention. Rev. Endocr. Metab. Disord..

[6] Bouillon R., Marcocci C., Carmeliet G., Bikle D., White J.H., Dawson-Hughes B., Lips P., Munns C.F., Lazaretti-Castro M., Giustina A. (2019). Skeletal and extraskeletal actions of vitamin D: Current evidence and outstanding questions. Endocr. Rev..

[7] Park C.Y., Shin S., Han S.N. (2024). Multifaceted Roles of Vitamin D for Diabetes: From Immunomodulatory Functions to Metabolic Regulations. Nutrients.

[8] Park C.Y., Han S.N. (2021). The role of vitamin D in adipose tissue biology: Adipocyte differentiation, energy metabolism, and inflammation. J. Lipid Atheroscler..

[9] Martineau A.R., Forouhi N.G. (2020). Vitamin D for COVID-19: A case to answer?. Lancet Diabetes Endocrinol..

[10] Kim H.K., Park C.Y., Han S.N. (2021). Nutrient modulation of viral infection-implications for COVID-19. Nutr. Res. Pract..

[11] Cashman K.D., Dowling K.G., Škrabáková Z., Gonzalez-Gross M., Valtueña J., De Henauw S., Moreno L., Damsgaard C.T., Michaelsen K.F., Mølgaard C. (2016). Vitamin D deficiency in Europe: Pandemic?. Am. J. Clin. Nutr..

[12] De-Regil L.M., Palacios C., Lombardo L.K., Peña-Rosas J.P. (2016). Vitamin D supplementation for women during pregnancy. Cochrane Database Syst. Rev..

[13] Liu X., Baylin A., Levy P.D. (2018). Vitamin D deficiency and insufficiency among US adults: Prevalence, predictors and clinical implications. Br. J. Nutr..

[14] Lee Y.A., Kim H.Y., Hong H., Kim J.Y., Kwon H.J., Shin C.H., Yang S.W. (2014). Risk factors for low vitamin D status in Korean adolescents: The Korea National Health and Nutrition Examination Survey (KNHANES) 2008–2009. Public Health Nutr..

[15] Pang Y., Kim O., Choi J.-A., Jung H., Kim J., Lee H., Lee H. (2021). Vitamin D deficiency and associated factors in south Korean childbearing women: A cross-sectional study. BMC Nurs..

[16] Korea Disease Control and Prevention Agency Korea National Health & Nutrition Examination Survey IX-1. https://knhanes.kdca.go.kr/knhanes/main.do.

[17] Oh K., Kim Y., Kweon S., Kim S., Yun S., Park S., Lee Y.K., Kim Y., Park O., Jeong E.K. (2021). Korea National Health and Nutrition Examination Survey, 20th anniversary: Accomplishments and future directions. Epidemiol. Health.

[18] Korean Food Composition Database 9.3. Rural Development Administration & National Institute of Agricultural Sciences: 2021. http://koreanfood.rda.go.kr/.

[19] Ross A.C., Manson J.E., Abrams S.A., Aloia J.F., Brannon P.M., Clinton S.K., Durazo-Arvizu R.A., Gallagher J.C., Gallo R.L., Jones G. (2011). The 2011 report on dietary reference intakes for calcium and vitamin D from the Institute of Medicine: What clinicians need to know. J. Clin. Endocrinol. Metab..

[20] Kim Y.S., Hwang J.H., Song M.R. (2018). The Association Between Vitamin D Deficiency and Metabolic Syndrome in Korean Adolescents. J. Pediatr. Nurs..

[21] Lee J.S., Kim Y.H. (2020). Vitamin D Status and Related Factors among Korean Stroke Survivors: A Nationwide Population-Based Study. J. Nutr. Sci. Vitaminol..

[22] Kim S., Lee G.W., Park C.Y. (2022). Older Korean men with inadequate vitamin D status have lower odds of radiologic osteoarthritis. Sci. Rep..

[23] Kim M.K., Baek K.H., Song K.H., Il Kang M., Park C.Y., Lee W.Y., Oh K.W. (2011). Vitamin D deficiency is associated with sarcopenia in older Koreans, regardless of obesity: The Fourth Korea National Health and Nutrition Examination Surveys (KNHANES IV) 2009. J. Clin. Endocrinol. Metab..

[24] Kang J.H., Kim S.S., Moon S.S., Kim W.J., Bae M.J., Choi B.G., Jeon Y.K., Kim B.H., Kim Y.K., Kim I.J. (2013). Adiposity in the Relationship between Serum Vitamin D Level and Insulin Resistance in Middle-Aged and Elderly Korean Adults: The Korea National Health and Nutrition Examination Survey 2008. Endocrinol. Metab..

[25] Scragg R., Sowers M., Bell C. (2004). Serum 25-hydroxyvitamin D, diabetes, and ethnicity in the Third National Health and Nutrition Examination Survey. Diabetes Care.

[26] Hadgu A., Yan F., Mayberry R. (2024). The Association Between Vitamin D Deficiency and Diabetes in Adult African Americans and Whites: An NHANES Study. J. Racial Ethn. Health Disparities.

[27] Kweon S., Kim Y., Jang M.J., Kim K., Choi S., Chun C., Khang Y.H., Oh K. (2014). Data resource profile: The Korea National Health and Nutrition Examination Survey (KNHANES). Int. J. Epidemiol..

[28] Jeon J., Lee S., Oh C. (2022). Age-specific risk factors for the prediction of obesity using a machine learning approach. Front. Public Health.

[29] Kim J.O.R., Jeong Y.S., Kim J.H., Lee J.W., Park D., Kim H.S. (2021). Machine Learning-Based Cardiovascular Disease Prediction Model: A Cohort Study on the Korean National Health Insurance Service Health Screening Database. Diagnostics.

[30] Oh T., Kim D., Lee S., Won C., Kim S., Yang J.S., Yu J., Kim B., Lee J. (2022). Machine learning-based diagnosis and risk factor analysis of cardiocerebrovascular disease based on KNHANES. Sci. Rep..

[31] Wu X., Park S. (2023). A Prediction Model for Osteoporosis Risk Using a Machine-Learning Approach and Its Validation in a Large Cohort. J. Korean Med. Sci..

[32] Yu A., Kim J., Kwon O., Oh S.-y., Kim J., Yang Y.J. (2014). Associations between serum 25-hydroxyvitamin D and consumption frequencies of vitamin D rich foods in Korean adults and older adults. Korean J. Community Nutr..

[33] Yoo K., Cho J., Ly S. (2016). Vitamin D Intake and Serum 25-Hydroxyvitamin D Levels in Korean Adults: Analysis of the 2009 Korea National Health and Nutrition Examination Survey (KNHANES IV-3) Using a Newly Established Vitamin D Database. Nutrients.

[34] Kim K.N., Lee J.S., Shim J.S., Yoon M.O., Lee H.S. (2023). Estimated dietary vitamin D intake and major vitamin D food sources of Koreans: Based on the Korea National Health and Nutrition Examination Survey 2016–2019. Nutr. Res. Pract..

[35] Guo J., He Q., Li Y. (2024). Machine learning-based prediction of vitamin D deficiency: NHANES 2001–2018. Front. Endocrinol..

[36] Lee E., Jang J.A., Kim J.-M. (2024). Eating habits and dietary supplement utilization according to food-related lifestyle among Korean adults: A cross-sectional study. Korean J. Community Nutr..

[37] Park H.A. (2018). Which Types of Dietary Supplements Are Used in Korea? Data from the 2015 Korea National Health and Nutrition Examination Survey. Korean J. Health Promot..

[38] Jeong H., Cho W.K., Jeong C.-E., Lee J.E. Assessment of excessive nutrient intake from health functional foods. Proceedings of the Korean Nutrition Society International Conference and Annual Meeting.

[39] Park J.H., Hong I.Y., Chung J.W., Choi H.S. (2018). Vitamin D status in South Korean population: Seven-year trend from the KNHANES. Medicine.

[40] Hamulka J., Jeruszka-Bielak M., Górnicka M., Drywień M.E., Zielinska-Pukos M.A. (2020). Dietary Supplements during COVID-19 Outbreak. Results of Google Trends Analysis Supported by PLifeCOVID-19 Online Studies. Nutrients.

[41] Zhao B., Yang S. (2024). Exploring the unique association between high-density lipoprotein cholesterol and vitamin D deficiency in adults aged 20–59: Findings based on the NHANES database. BMC Endocr. Disord..

[42] Ponda M.P., Huang X., Odeh M.A., Breslow J.L., Kaufman H.W. (2012). Vitamin D may not improve lipid levels: A serial clinical laboratory data study. Circulation.

[43] Alkhatatbeh M.J., Amara N.A., Abdul-Razzak K.K. (2019). Association of 25-hydroxyvitamin D with HDL-cholesterol and other cardiovascular risk biomarkers in subjects with non-cardiac chest pain. Lipids Health Dis..

[44] Wakayo T., Belachew T., Vatanparast H., Whiting S.J. (2015). Vitamin D deficiency and its predictors in a country with thirteen months of sunshine: The case of school children in central Ethiopia. PLoS ONE.

[45] Griffin T.P., Wall D., Blake L., Griffin D.G., Robinson S., Bell M., Mulkerrin E.C., O’Shea P.M. (2020). Higher risk of vitamin D insufficiency/deficiency for rural than urban dwellers. J. Steroid Biochem. Mol. Biol..

[46] Peters B.S.E., Verly E., Marchioni D.M.L., Fisberg M., Martini L.A. (2012). The influence of breakfast and dairy products on dietary calcium and vitamin D intake in postpubertal adolescents and young adults. J. Hum. Nutr. Diet..

[47] Fagnant H.S., Lutz L.J., Nakayama A.T., Gaffney-Stomberg E., McClung J.P., Karl J.P. (2022). Breakfast skipping is associated with vitamin D deficiency among young adults entering initial military training. J. Acad. Nutr. Diet..

[48] Heymsfield S.B., Arteaga C., McManus C., Smith J., Moffitt S. (1983). Measurement of muscle mass in humans: Validity of the 24-hour urinary creatinine method. Am. J. Clin. Nutr..

[49] Patel S.S., Molnar M.Z., Tayek J.A., Ix J.H., Noori N., Benner D., Heymsfield S., Kopple J.D., Kovesdy C.P., Kalantar-Zadeh K. (2013). Serum creatinine as a marker of muscle mass in chronic kidney disease: Results of a cross-sectional study and review of literature. J. Cachexia Sarcopenia Muscle.

[50] Ureña-Torres P., Metzger M., Haymann J.P., Karras A., Boffa J.-J., Flamant M., Vrtovsnik F., Gauci C., Froissart M., Houillier P. (2011). Association of kidney function, vitamin D deficiency, and circulating markers of mineral and bone disorders in CKD. Am. J. Kidney Dis..

[51] de Boer I.H., Ioannou G.N., Kestenbaum B., Brunzell J.D., Weiss N.S. (2007). 25-Hydroxyvitamin D levels and albuminuria in the Third National Health and Nutrition Examination Survey (NHANES III). Am. J. Kidney Dis..

[52] Gupta S., Goyal P., Feinn R.S., Mattana J. (2019). Role of Vitamin D and Its Analogues in Diabetic Nephropathy: A Meta-analysis. Am. J. Med. Sci..

[53] Fakhoury M., Levy R., Melamed M.L. (2019). Vitamin D deficiency and kidney hyperfiltration: A mechanism of kidney injury?. Ann. Transl. Med..

[54] Agarwal R., Hynson J.E., Hecht T.J., Light R.P., Sinha A.D. (2011). Short-term vitamin D receptor activation increases serum creatinine due to increased production with no effect on the glomerular filtration rate. Kidney Int..

[55] Jones P., Lucock M., Veysey M., Beckett E. (2018). The Vitamin D⁻Folate Hypothesis as an Evolutionary Model for Skin Pigmentation: An Update and Integration of Current Ideas. Nutrients.

[56] Eloranta J.J., Zaïr Z.M., Hiller C., Häusler S., Stieger B., Kullak-Ublick G.A. (2009). Vitamin D3 and its nuclear receptor increase the expression and activity of the human proton-coupled folate transporter. Mol. Pharmacol..

[57] Visentin M., Diop-Bove N., Zhao R., Goldman I.D. (2014). The intestinal absorption of folates. Annu. Rev. Physiol..

[58] Kim S.H., Oh M.K., Namgung R., Park M.J. (2014). Prevalence of 25-hydroxyvitamin D deficiency in Korean adolescents: Association with age, season and parental vitamin D status. Public Health Nutr..

[59] Yu H.J., Kwon M.J., Woo H.Y., Park H. (2016). Analysis of 25-Hydroxyvitamin D Status According to Age, Gender, and Seasonal Variation. J. Clin. Lab. Anal..

[60] Lee J., Won Woo H., Kim J., Shin M.H., Koh I., Youl Choi B., Kyung Kim M. (2021). Independent and interactive associations of season, dietary vitamin D, and vitamin D-related genetic variants with serum 25(OH)D in Korean adults aged 40 years or older. Endocr. J..

[61] Mason R.S., Rybchyn M.S., Abboud M., Brennan-Speranza T.C., Fraser D.R. (2019). The Role of Skeletal Muscle in Maintaining Vitamin D Status in Winter. Curr. Dev. Nutr..

[62] Rybchyn M.S., Abboud M., Puglisi D.A., Gordon-Thomson C., Brennan-Speranza T.C., Mason R.S., Fraser D.R. (2020). Skeletal Muscle and the Maintenance of Vitamin D Status. Nutrients.

