# Supplementary material for: Discovering Vitamin-D-Deficiency-Associated Factors in Korean Adults Using KNHANES Data Based on an Integrated Analysis of Machine Learning and Statistical Techniques
Source: Nutrients. 2025 Feb 8;17(4):618. doi: 10.3390/nu17040618 (PMC11858147; doi:10.3390/nu17040618)
Supplement: Supplementary file 1 [file nutrients-17-00618-s001.zip › nutrients-3435395-supplementary.pdf]

Table S1. General characteristics of the participants aged 19-64y

	Men (n=1424)			Women (n=1895)		
	**VD deficient (n= 729)**	**VD sufficient (n= 695)**	***p* value***	**VD deficient (n= 857)**	**VD sufficient (n= 1038)**	***p* value***
Age, y	39.5 ± 0.5	45.2 ± 0.6	<0.001	38.7 ± 0.5	46.9 ± 0.5	<0.001
Household income, %			0.077			0.359
Low	69 (9)	59 (8)		78 (9)	108 (9)	
Middle-low	142 (19)	127 (16)		225 (24)	227 (20)	
Middle-high	245 (35)	213 (31)		275 (32)	339 (34)	
High	272 (37)	296 (45)		278 (35)	364 (37)	
Current alcohol consumer, %	512 (70)	483 (69)	0.793	449 (54)	482 (46)	0.005
Current smoker, %	254 (34)	205 (28)	0.053	58 (7)	45 (4)	0.010
Regular aerobic exercise, %	376 (34)	348 (39)	0.946	420 (25)	476 (33)	0.224
Body mass index, kg/m^2	25.4 ± 0.2	25.1 ± 0.2	0.130	23.3 ± 0.2	22.9 ± 0.1	0.083
Total energy intake, kcal/d	2191 ± 36	2194 ± 32	0.948	1598 ± 57	1545 ± 21	0.122
Vitamin D intake, µg/d	3.2 ± 0.2	3.7 ± 0.3	0.095	2.7 ± 0.2	2.9 ± 0.2	0.650
Blood 25(OH)D3 level, ng/mL	13.6 ± 0.2	28.6 ± 0.3	<0.001	13.4 ± 0.2	30.4 ± 0.3	<0.001

Data are expressed as means ± SE for continuous variables or number (%) for categorical variables.

*Differences were determined via ANOVA for continuous variables or Rao-Scott chi-square tests for categorical variables.

Table S2. General characteristics of the participants aged ≥65y

	Men (n= 671)			Women (n= 831)		
	VD deficient (n= 226)	VD sufficient (n= 445)	*p* value*	VD deficient (n= 222)	VD sufficient (n= 609)	*p* value*
Age, y	73.5 ± 0.4	72.2 ± 0.3	0.037	73.2 ± 0.5	72.5 ± 0.3	0.192
Household income, %			0.365			0.418
Low	100 (40)	165 (34)		119 (51)	297 (45)	
Middle-low	63 (29)	156 (37)		54 (23)	182 (31)	
Middle-high	40 (21)	73 (17)		31 (16)	79 (15)	
High	23 (11)	51 (13)		18 (10)	47 (10)	
Current alcohol consumer, %	112 (52)	250 (59)	0.087	35 (17)	99 (16)	0.488
Current smoker, %	39 (18)	93 (19)	0.929	2 (1)	11 (2)	0.759
Regular aerobic exercise, %	70 (34)	153 (39)	0.335	49 (25)	167 (33)	0.036
Body mass index, kg/m^2	24.0 ± 0.2	24.0 ± 0.1	0.943	24.8 ± 0.3	24.2 ± 0.2	0.076
Total energy intake, kcal/d	1882 ± 51	1893 ± 34	0.871	1412 ± 43	1426 ± 31	0.791
Vitamin D intake, µg/d	2.6 ± 0.3	3.6 ± 0.3	0.035	2.0 ± 0.3	2.4 ± 0.2	0.207
Blood 25(OH)D3 level, ng/mL	14.4 ± 0.4	30.0 ± 0.5	<0.001	14.3 ± 0.3	33.7 ± 0.5	<0.001

Data are expressed as means ± SE for continuous variables or number (%) for categorical variables.
*Differences were determined via ANOVA for continuous variables or Rao-Scott chi-square tests for categorical variables.

Table S3. Association between serum 25(OH)D3 level and 14 VDD-associated continuous variables identified through machine learning by age and sex (including standard error values).

Continuous Variables	19–64 years	Model 1*		Model 2†		≥ 65 years	Model 1*		Model 2†	
		β (SE)	*p*-value	β (SE)	*p*-value		β (SE)	*p*-value	β (SE)	*p*-value
Blood urea nitrogen	Men	0.386 (0.071)	<0.001	0.398 (0.075)	<0.001	Men	0.297 (0.109)	<0.001	0.313 (0.109)	0.005
	Women	0.105 (0.074)	0.16	0.121 (0.072)	0.09	Women	0.179 (0.082)	0.03	0.160 (0.084)	0.06
Waist circumference	Men	-0.185 (0.069)	0.008	-0.190 (0.074)	0.01	Men	0.012 (0.113)	0.92	0.040 (0.112)	0.72
	Women	-0.048 (0.083)	0.56	-0.028 (0.086)	0.75	Women	-0.077 (0.120)	0.52	-0.116 (0.133)	0.39
Serum HDL cholesterol	Men	0.075 (0.021)	0.001	0.067 (0.022)	0.003	Men	0.041 (0.035)	0.25	0.044 (0.038)	0.25
	Women	0.042 (0.018)	0.02	0.041 (0.019)	0.03	Women	0.071 (0.041)	0.09	0.052 (0.039)	0.18
Urinary sodium	Men	-0.010 (0.005)	0.04	-0.009 (0.005)	0.08	Men	-0.016 (0.011)	0.12	-0.016 (0.011)	0.15
	Women	-0.017 (0.006)	0.004	-0.015 (0.005)	0.005	Women	-0.037 (0.012)	0.002	-0.037 (0.013)	0.004
Blood creatinine	Men	8.16 (1.71)	<0.001	8.16 (1.95)	<0.001	Men	-1.926 (1.993)	0.34	-1.97 (1.94)	0.31
	Women	4.93 (2.32)	0.04	5.19 (2.27)	0.02	Women	1.286 (2.834)	0.65	1.17 (2.86)	0.68
Folate intake	Men	0.002 (0.002)	0.32	0.001 (0.002)	0.62	Men	-0.001 (0.003)	0.82	-0.001 (0.003)	0.68
	Women	0.001 (0.002)	0.55	0.002 (0.002)	0.49	Women	-0.001 (0.004)	0.89	-0.001 (0.004)	0.79
Average daily sitting time	Men	0.010 (0.011)	0.37	0.002 (0.057)	0.98	Men	-0.016 (0.020)	0.42	-0.065 (0.037)	0.09
	Women	0.015 (0.013)	0.27	-0.061 (0.063)	0.34	Women	-0.026 (0.022)	0.24	-0.032 (0.048)	0.50
	Men	-0.023 (0.008)	0.003	-0.022 (0.008)	0.006	Men	-0.035 (0.012)	0.006	-0.033 (0.012)	0.009

Fasting plasma glucose	Women	-0.018 (0.014)	0.20	-0.012 (0.014)	0.39	Women	-0.008 (0.024)	0.75	-0.000 (0.024)	1.00
SGOT	Men	0.009 (0.016)	0.56	-0.004 (0.014)	0.79	Men	0.012 (0.054)	0.83	-0.008 (0.057)	0.89
	Women	0.039 (0.026)	0.14	0.032 (0.024)	0.18	Women	0.035 (0.046)	0.45	0.036 (0.045)	0.42
Water intake	Men	0.002 (0.001)	0.003	0.001 (0.001)	0.02	Men	0.003 (0.001)	0.006	0.003 (0.001)	0.02
	Women	0.001 (0.001)	0.02	0.002 (0.001)	0.02	Women	0.002 (0.002)	0.25	0.002 (0.002)	0.27
Weight	Men	0.086 (0.046)	0.06	0.084 (0.048)	0.08	Men	0.163 (0.093)	0.08	0.209 (0.088)	0.02
	Women	0.007 (0.060)	0.90	0.009 (0.065)	0.90	Women	0.047 (0.120)	0.70	-0.064 (0.126)	0.61
Hemoglobin	Men	-0.364 (0.247)	0.14	-0.280 (0.247)	0.26	Men	0.152 (0.420)	0.72	0.055 (0.393)	0.89
	Women	0.778 (0.249)	0.002	0.791 (0.264)	0.003	Women	0.157 (0.455)	0.73	0.392 (0.500)	0.43
Urinary creatinine	Men	-0.010 (0.003)	0.001	-0.009 (0.003)	0.002	Men	-0.009 (0.008)	0.26	-0.007 (0.008)	0.38
	Women	-0.014 (0.004)	<0.001	-0.014 (0.004)	0.001	Women	-0.029 (0.011)	0.009	-0.036 (0.010)	0.001
Red blood cells	Men	-0.973 (0.721)	0.18	-0.762 (0.746)	0.31	Men	-0.120 (1.220)	0.92	-0.228 (1.153)	0.84
	Women	-0.536 (0.875)	0.54	-0.412 (0.897)	0.65	Women	-0.414 (1.306)	0.75	0.398 (1.384)	0.77

This analysis was conducted using multiple regression analysis between serum 25(OH)D3 level and each variable identified through machine learning with adjustment.

* Model 1: adjusted for age, body mass index, and total energy intake.

† Model 2: model 1 plus adjustments for household income, alcohol consumption, smoking, aerobic exercise, and vitamin D intake.

25(OH)D_3, 25-hydroxyvitamin D3; HDL, high-density lipoprotein; SE, standard error; SGOT, serum glutamic–oxaloacetic transaminase; VD, vitamin D; VDD, vitamin D deficiency.
